# Interaction between respiration and central motor control in autonomic cardiac regulation

**DOI:** 10.1016/j.jphyss.2025.100054

**Published:** 2025-12-23

**Authors:** Pauline Doussineau, Antoine Mariani, Laurent Reale, Chantal Verkindt, Florian Chouchou

**Affiliations:** University of La Réunion, UFRSHE, IRISSE Laboratory (EA4075), Le Tampon, France

**Keywords:** Autonomic nervous system, Physical activity, RR variability, Movement, Respiration, Parasympathetic regulation

## Abstract

Adaptations in cardiac autonomic regulation induced by physical training are central to the health benefits of exercise. We investigated the interaction between central motor command, breathing rate, and expiration timing during an isolated joint movement. Fifteen volunteers (30.9 ± 7.2 years; 8 women) performed twelve 3-min leg extension tasks in a seated position under three randomized conditions: movement type (active vs. passive), breathing pattern (spontaneous, 12 or 6 cycles/min), and expiration timing (during extension vs. return). RR intervals and their variability were analyzed. Voluntary motor control reduced indices of autonomic and parasympathetic regulation (*p < 0.05*), whereas slow-paced breathing enhanced them. However, motor control exerted a dominant influence over cardiac autonomic regulation (*p < 0.001*), regardless of breathing rate. Expiration during the return phase was associated with greater parasympathetic activity (*p < 0.05*). These results highlight the central role of motor command in autonomic modulation and suggest that emphasizing expiration during recovery may help preserve parasympathetic regulation.

## Highlights

• Voluntary motor command exerts a stronger effect on cardiac autonomic control than respiration.
• Slow-paced breathing enhances parasympathetic activity, but is disrupted by active movement.
• Expiration during the recovery phase of movement preserves parasympathetic regulation.

## Introduction

Autonomic regulation undergoes significant changes during cardiovascular acute responses to exercise and adaptations to training programs, particularly aerobic training [1]. These cardiovascular changes are underpinned by the predominance of sympathetic activity during exercise according to the intensity, environment and duration of exercise; while training programs will favor parasympathetic regulation and slow cardiac rhythmic activity at rest [2], [3]. These exercise-induced modulations in cardiac autonomic control play a key role in mediating the broader health benefits associated with physical activity [4].

The main mechanism supporting this autonomic activation during exercise relies on metaboreflexes [5], [6] but this is not the only mechanism of cardiovascular regulation active during exercise. This cardiovascular response could be initiated by a central command which involves higher brain network including motor network [7], as well as cognitive and emotions brain networks that activate parallel circuits controlling locomotor, cardiovascular and ventilatory functions [8], [9]. Its contribution seems more modest with intense exercise, but it could contribute more meaningfully to the cardiovascular adaptations of exercise at lower intensity or in the initiation of movements [10], [11]. However, muscular afferent input may play a role in these cardiovascular autonomic adaptations: passive stretch in healthy volunteers could increase cardiac rhythms and inhibit parasympathetic cardiac control [12].

Furthermore, considering the significant sedentary lifestyle of modern society [13], many physical practices are offered, in particular to patients suffering from chronic diseases who rely on exercises of modest intensity. These practices can also be based on slow-paced breathing, alone or combined with movements, which are practiced for their potential health benefits [14]. More recently, studies have proposed that slow-paced breathing at 6 cycles/min can promote parasympathetic activity through resonance phenomena within the cardiorespiratory system [15]. Numerous studies had clearly shown respiratory control effect on cardiac autonomic regulation where the slow-paced breathing and the expiration phase promotes parasympathetic tone [16], [17].

Finally, interaction between motor and respiration controls is poorly documented [18]. Al-Ani and colleagues reported that during expiration, where cardiac parasympathetic activity increased, the amplitude of the cardiac response to electrical evoked arm flexion was greater than that performed during inspiration, indicating that skeletal muscle afferent may inhibit cardiac respiratory driven parasympathetic tone [19]. Thus, the synchronization between the motor phase and the expiration phase could increase the autonomic response to physical exercise, in particular by inhibiting parasympathetic control.

Understanding how physical exercise solicits the autonomic nervous system is fundamental to maximizing its protective effects. Here, we studied the interaction between central command, respiratory control and expiration timing during movement in autonomic cardiac control. For this purpose, in the present study, the interaction between central command and respiratory control (respiratory rate and expiratory time) in cardiac autonomic regulation will be examined under simple conditions, similar to certain practices such as yoga or traditional Chinese gymnastics — an approach that, to our knowledge, has not been previously investigated. Accordingly, we hypothesized that while slow-paced breathing promotes parasympathetic control, voluntary motor control enhances sympathetic activity regardless of respiratory control.

## Methods

### Subjects

Fifteen healthy volunteers (8 women, age: 30.9 ± 7.2 years, body mass index: 23.2 ± 2.45 kg/m²) participated in this experiment. They were free from any known cardiorespiratory abnormalities, neurological or psychiatric disease, and none of them was on any medication. They were asked to refrain from practicing physical activity 24 h before the experiment and could take coffee or tea before. They were recruited through poster advertisements at various faculties and departments of the University of La Réunion. The study was approved by the Ethics Committee (IRB00012476–2022–24–06–193 CERSTAPS) and performed under informed consent according to the Declaration of Helsinki.

### Experimental protocol

**Fig. 1:**
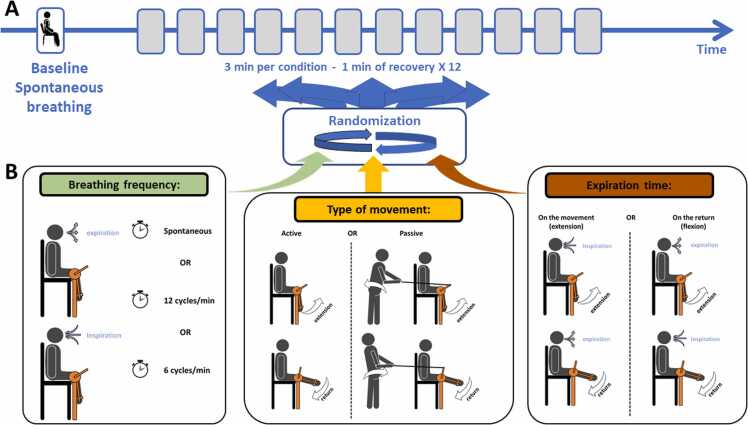
Experimental protocol. A) after the baseline at spontaneous breathing, subjects underwent 12 experimental conditions. Each condition lasted 3 min and was separated by 1-min recovery; B) Three experimental effects were randomized: breathing control (spontaneous breathing, at 6 cycles/min or 12 cycles/min), type of movement (passive or active movement), and time of breathing (expiration or inspiration on active phase of movement); C) Schematic representation of the different experimental conditions.

The protocol consisted of 3-min basal recording followed by 12 randomized 3-min experimental conditions separated by 1-min recovery (Fig. 1). Three experimental conditions were randomized in a balanced fashion between 3 experimental effects including: 1) the type of movement (passive or active); 2) the breathing frequency (3 different conditions: spontaneous, 12 respiratory cycles/minute (min) or 6 respiratory cycles/min); and 3) the expiration timing (on the return – flexion or on the movement – extension).

Each volunteer took his/her place on a custom-built apparatus design to study leg extensions. The setup consisted of a chair and a vertical board attached to the right ankle, with its axis of rotation positioned beneath the knee. Participants were seated with their back straight, gaze directed forward, legs parallel, and forearms resting on their thighs [20]. The volunteers performed a leg extension, limited at 90 ° (°) extension and beginning at 90° flexion. During passive mobilizations, the experimenter evoked the extension by using a pulley system. Volunteers were not allowed to talk during experimental conditions.

All movement and paced breathing frequency were synchronous with a metronome developed on a lab computer. In order to respect the normal rhythm of breathing, one-third of the respiratory cycle is allocated to inspiration and the remaining two-thirds to expiration. In order to better understand the flexion and extension phases coupled with the inspiration and expiration phases, volunteers were asked to start the extension movement and adequate breathing at the start of the recording. For spontaneous breathing when muscle contraction is the only one imposed, the metronome was used only for leg extensions. Under spontaneous breathing conditions, only the movements were synchronized at 6 or 12 cycles per minute. Under this experimental condition, participants were asked to concentrate only on the frequency of their movements, while avoiding any voluntary control of respiration.

### Recording procedure

Electrocardiography (ECG), respiration, neuromuscular control, and leg movements were continuously recorded. A 3-lead ECG was applied to the chest and rib areas in a Lead II position. The data were recorded at a sampling rate of 512 Hz and recorded using Movesense® recording system associated with sensors (Suunto, Vantaa, Finland). The lead with the greatest R wave amplitude was subsequently analyzed. To control the respect of the metronome frequency, respiration was monitored by a piezoelectric belt for chest movements at a sampling frequency of 500 Hz (National Instruments data acquisition system, USA). To control the absence of muscular activation during passive movement condition, neuromuscular activity was monitored by surface electromyography (EMG) using 6 electrodes (MAZET Electronic, France) on the right quadriceps and right hamstring, one ground electrode for each muscle was placed on bone tissue. EMG activity was collected at a frequency of 500 Hz. Finally, we measured the range of extension/flexion using a goniometer (Mazet Electronic, France) placed on the chair and measured the platform rotation movement. The goniometer output has a resolution of 1° and the sampling rate was at 500 Hz. Finally, all the measurement tools were resampled at 500 Hz using data acquisition software on LabView® (Austin, Texas, USA).

### Data analysis

#### ECG preprocessing

**Fig. 2:**
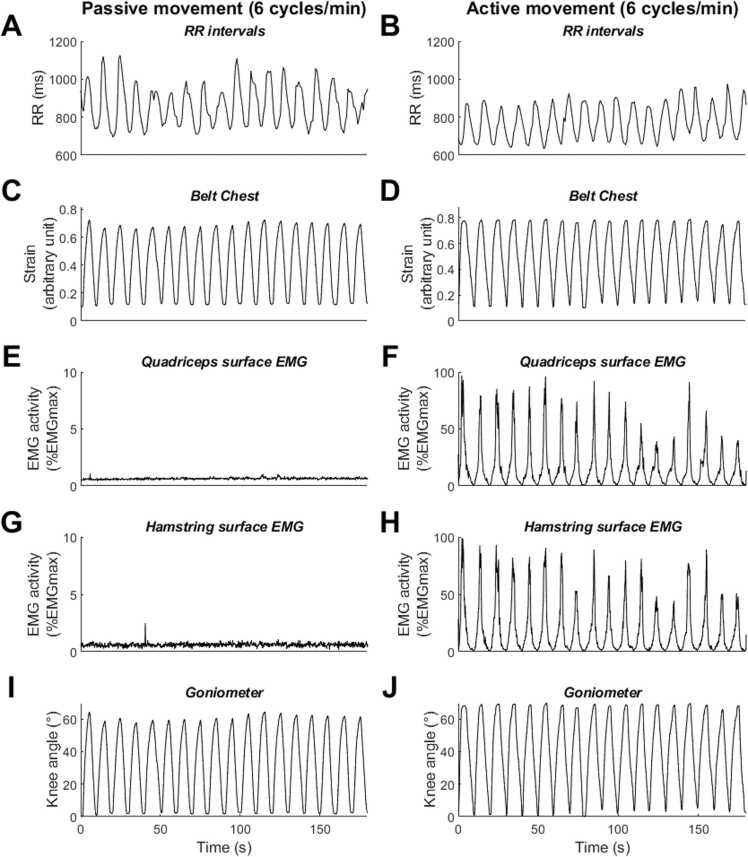
Representative example of passive and active movement conditions. A few minutes of recordings illustrate changes in RR intervals (**A**, **B**), respiration (chest belt; **C**, **D**), quadriceps electromyography (EMG; **E**, **F**), hamstring electromyography (**G**, **H**), and movement (goniometer; **I**, **J**) according to passive (**A**, **C**, **E**, **G**, **I**) and active (**B**, **D**, **F**, **H**, **J**) experimental conditions. This example illustrates the fluctuation of RR intervals across the respiratory cycle, demonstrating respiratory sinus arrhythmia. It also reveals faster RR intervals during the active movement condition compared to the passive condition.

ECG signals were subjected to peak-to-peak analysis to detect R waves to calculate the RR intervals (RRI) signal using a dedicated software [21]. Initial automatized extraction of RRI signal from ECG data was subsequently checked by visual inspection. In the presence of undetected isolated QRS, the RRI signal was manually corrected. In the case of ectopic beats or artifacts, a cubic spline interpolation was used to correct RRI signal [22]. RRI were expressed as a function of time (Fig. 2).

#### RRI variability analysis

Time- and frequency-domain RRI variability analysis was done using a dedicated software [21]. Spectral analysis was performed by fast Fourier transform, calculated on sets of RRI during each experimental period for each subject. Fourier transform was applied to a time series of consecutive 256 points during the 3 min of experimental conditions. Means of these spectrums were calculated for each subject and each condition. RRI variability indices were calculated as recommended by a task force of the European Society of Cardiology and the North American Society of Pacing and Electrophysiology [23]. Total power (Ptot) (0.00–0.40 Hz) represents the overall autonomic control; high frequency power (HF) of RRI (0.15–0.40 Hz) is known to represent parasympathetic activity related to breathing control; low frequency power (LF) of RRI (0.04–0.15 Hz) represents both parasympathetic and sympathetic activities [24]. Normalized indices (normalized LF, normalized HF, LF/HF ratio) were not used in this study, while maintaining a respiratory rate in the HF is recommended to interpret the normalized indices [25]. Means of these spectra were calculated for each condition.

For time-domain, standard deviation of normal-to-normal intervals (SDNN), standard deviation of the root mean square of successive difference (RMSSD), and percentage of successive RRI differing by more than 50 ms (pNN50) were studied. SDNN was chosen as it represents overall autonomic control, whereas RMSSD and pNN50 are recognized indices of parasympathetic activity [23].

#### Other parameters: respiratory, neuromuscular and leg controls

To ensure that the experimental conditions were respected (Fig. 2), breathing, neuromuscular control and range of motion were analyzed using Matlab® software (The MathWorks Inc., Natick, MA, USA). Respiration was studied using a piezo sensor. Signal was low-pass filtered at 10 Hz and analysis consisted of calculating the respiratory rate by cycles/min for each experimental condition [26]. Additionally, movements were studied using a goniometer, also low-pass filtered at 10 Hz, and range (in degree) and frequency (in cycles/min) of movement were calculated [20]. Finally, the EMG signal up to 500 Hz was studied to be sure that there was no muscle activation during the experimental condition without central command. Neuromuscular analysis consisted of calculating root mean square (RMS) value on sliding windows of 100-ms, expressed as the percentage of EMG activation in comparison with maximal EMG activation during the experimental recording for each subject [20].

### Statistical analysis

Statview® (SAS Institute, Inc., Cary, NC, USA) and SPSS® softwares (IBM, Armonk, NY, USA) were used for statistical analyses. An a priori power analysis was performed using *G*Power* (G*Power 3.1.9.7, Heinrich Heine University Düsseldorf, Germany), based on the results reported by Al-Ani et al. [19]. We estimated that a sample of 10 subjects would be necessary to detect an effect size of 0.7 — a value deliberately overestimated to prevent potential subject loss (data acquisition problems, a posteriori exclusion, etc.), which did not occur. All data were submitted to repeated measures analysis of variance (RM-ANOVA) with four within factors, central command (passive vs. active movements), respiratory control (spontaneous breathing, 12 cycles/min vs. 6 cycles/min), and expiration timing (expiration on return vs. expiration on movement). The Greenhouse–Geisser correction was applied to adjust for violations of the sphericity assumption. Tukey post hoc test was performed when appropriate. Significance was taken to be a *p* value of < 0.05.

## Results

### Autonomic cardiac control

**Fig. 3:**
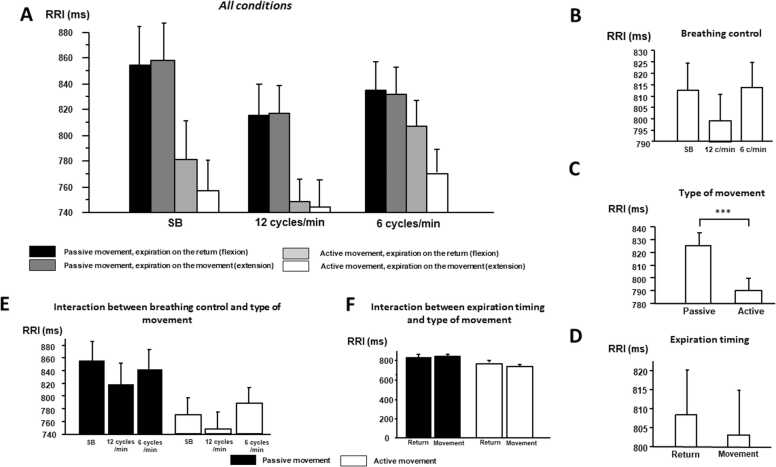
**Changes in RRI according to breathing control, type of movement and the moment of expiration (mean ± standard error). A)** All conditions; **B)** breathing control; **C)** movement type; **D)** expiration timing; and **E)** interactions between breathing control and type of movement F**)** between expiration timing and type of movement. RRI: RR intervals, SB: spontaneous breathing.

**Fig. 4:**
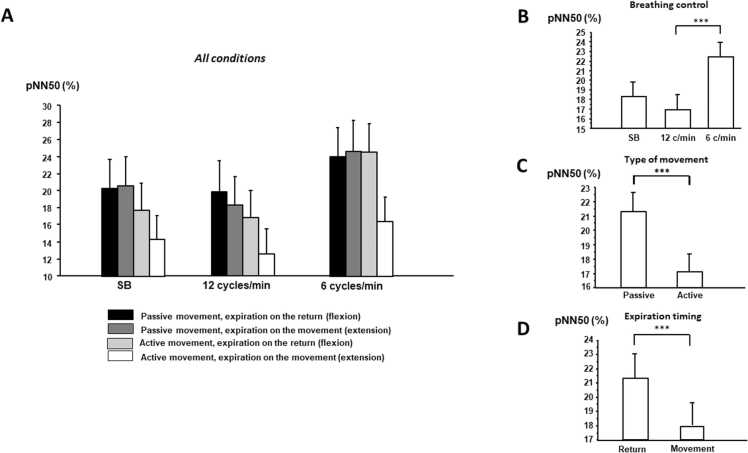
**Changes in pNN50 according to breathing control, the type of movement and the moment of expiration (mean ± standard error). A)** All conditions; **B)** breathing control; **C)** movement type and **D)** expiration timing. pNN50: percentage of successive RRI differing by more than 50 ms, SB: spontaneous breathing.

**Fig. 5:**
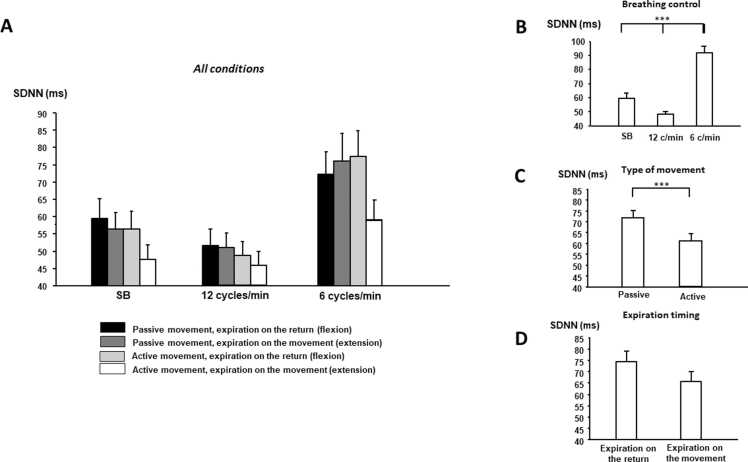
**Changes in SDNN according to breathing control, the type of movement and the moment of expiration (mean ± standard error). A)** All conditions; **B)** breathing control; **C)** movement type and **D)** expiration timing. SDNN: standard deviation of normal-to-normal intervals, SB: spontaneous breathing.

Results of autonomic cardiac parameters experimental conditions are presented in Fig. 3, Fig. 4, Fig. 5 and Table 1.

**Table 1:** Cardiac, autonomic, neuromuscular and movement parameters according to each experimental condition: breathing control (spontaneous breathing, at 6 cycles/min or 12 cycles/min), type of movement (passive or active movement), and time of breathing (expiration or inspiration on active phase of movement). RRI: RR intervals, SDNN: standard deviation of normal-to-normal intervals, RMSSD: standard deviation of the root mean square of successive difference, pNN50: percentage of successive RRI differing by more than 50 ms, Ptot: total power, LF: low frequency, HF: high frequency, EMG: electromyography. The statistical section reports the F and p values from the ANOVA for each effect: movement type (passive or active), breathing pattern (spontaneous, 12 or 6 cycles per minute), expiratory timing (inspiration or expiration during the return phase), and their interactions. The numbers 1, 2, and 3 correspond respectively to the experimental factors — movement type, breathing pattern, and expiratory timing — and are used to save space in the table columns.

RRI mainly decreased during active movement in comparison with passive (ANOVA, p < 0.001, post hoc test: p < 0.05). RRI also changed according to respiration pattern (ANOVA, p < 0.001) but without any significant post hoc tests. Interactions between movements, respiratory pattern and expiration timing appeared (Table 1).

Active movement induced a decrease in Ptot (p = 0.028), SDNN (p = 0.021) and in LF power (p = 0.043) as well as in pNN50 (p = 0.004) and tended toward a significant level in RMSSD (p = 0.077). Respiratory control also changed autonomic control: LF and Ptot increased at 6 cycles/min in comparison with 12 cycles/min and spontaneous breathing (post hoc analysis: p < 0.05); whereas HF power decreased at 6 cycles/min in comparison with 12 cycles/min. Concerning time domain, SDNN increased at 6 cycles/min in comparison with 12 cycles/min and spontaneous breathing (post hoc analysis: p < 0.05), and pNN50 and RMSSD increased at 6 cycles/min in comparison with 12 cycles/min (post hoc analysis: p < 0.05). Finally, expiration timing also impacted cardiac autonomic control: all time and frequency parameters were high when expiration accompanied return rather than movement (Table 1). Decreases in Ptot, LF power, SDNN, pNN50, and RMSSD due to active movement are higher when expiration timing accompanied movement (see Table 1).

These results illustrate that while voluntary respiratory or motor changes both influence cardiac autonomic regulation, the impact of voluntary motor control on cardiac control is greater than that of respiratory modulations.

### Other parameters: respiratory, neuromusclar and leg controls

Results of respiratory, neuromuscular and leg controls parameters are presented in Table 1. Active movement was accompanied by quadriceps and tibial hamstring EMG activations, not observed during passive movement. No difference appeared in respiratory frequency (piezo sensor, p = NS) and leg extension between passive and active movements (goniometer extension, p = NS). As expected, only changes in experimental respiratory conditions had an effect on respiratory frequency, where the latter was lower in the slow-paced breathing (6 cycles/min condition) than in the other two conditions (for both: p < 0.05), as well as in paced breathing at 12 cycles/min and spontaneous respiration control (p < 0.05), confirming that subjects adhere to the instructions. Neither movement nor activation of the tibial hamstring differs (p = NS). ANOVA of quadriceps EMG shows significant differences for the respiration effect but not post-hoc analyses. Finally, expiration on return was associated with a shorter leg movement (delta: 1.5°, p < 0.05).

These results indicate that participants adhered well to the respiratory and motor instructions.

## Discussion

The autonomic nervous system contributes to cardiovascular adaptions to exercise, characterized by decreased cardiac parasympathetic control for low-intensity exercise, and increased sympathetic tone for higher intensities [27], [28]. Here, we illustrated that: 1) while slow-paced breathing improves parasympathetic and overall autonomic regulation and central motor command decreases parasympathetic autonomic regulation, the impact of voluntary motor control on cardiac control is greater than that of respiratory modulations during a simple, isolated joint movement; and 2) expiration during the return of dynamic exercise may preserve parasympathetic regulation. Given that parasympathetic activity protects against cardiac rhythm disorders [29], we propose that engaging in rhythmic physical exercise, synchronizing the passive phase with expiration and by promoting a slow-paced breathing that enhance parasympathetic regulation during exercise.

### Slow-paced breathing disturbed by central motor command

Regarding the respiratory control, the implication of breathing in autonomic control – respiratory sinus arrhythmia - is well known [30], manifesting as RRI decrease during inspiration, and increasing during expiration. Previous studies reported during slow-paced breathing an increase in overall autonomic tone and parasympathetic control [31], [32], [33], [34], [35]. Recently, Laborde et al. (2022) showed in a large meta-analysis the impact of this type of slow-paced breathing practice on autonomic cardiac control, particularly the improvement of parasympathetic cardiac control during, immediately after, and in response to a program of several sessions in healthy subjects and patients suffering from chronic diseases [36]. In the present study, we observed that acute slow-paced breathing immediately favors parasympathetic control compared to pace-controlled or spontaneous breathings; but this simple, isolated joint active movement accelerated heart rhythm and reduced parasympathetic control whatever the respiratory control, including slow-paced breathing at 6 cycles/min. Consistently with ours results, [37] observed that light exercise on a cycle ergometer induces greater autonomic cardiac changes than slow-paced breathing. These autonomic-motor responses occur regardless of respiratory slow-paced, normal-paced or spontaneous breathings. These results and ours highlighted a primary contribution of ‘central command’ [38], assuming a direct action of locomotor centers on the medullary neurons of the respiratory and cardiovascular centers [39].

Consistently, animal studies have identified cortical networks that project to the autonomic structures of the brainstem: transneuronal transport of virus identified three major cerebral networks having multisynaptic connections with the adrenal medulla [40]. The largest influence originates from the sensorimotor network that includes all motor preparation and movement execution from the frontal regions (including the primary motor cortex (M1), the premotor areas, the supplementary motor area, the cingulate motor, as well as regions of somatosensory cortex and posterior parietal cortex). More recently, Gordon et al. [41] using functional magnetic resonance imaging methods in humans, observed that the homunculus in M1 is interrupted by regions with distinct and dense connectivity to other brain networks, including the cingulo-insular network, playing a role in central autonomic regulation [42]. Collectively, these findings highlight the primary functional and anatomical hierarchy of central command in autonomic regulation during exercise, likely acting to proactively adjust cardiovascular autonomic responses to anticipated physiological demands.

### Expiration on motor control or return during repetitive exercise

Finally, we observed that expiration during the return of dynamic exercise may preserve parasympathetic regulation. Parasympathetic control exerts a protective effect on numerous physiological processes [43]. It is commonly linked to a decreased risk of morbidity and mortality, whether in cardiovascular [44], metabolic [45], or other health contexts [46]. Specifically, parasympathetic activity guards against cardiac rhythm disorders [29]. During exercise, heightened sympathetic stimulation can induce arrhythmias, especially among patients with cardiovascular diseases [47].

Al-Ani and colleagues (1997) reported that during expiration, when cardiac parasympathetic activity was increased, the amplitude of the RRI response to electrically evoked arm flexion was higher than that obtained when contractions were performed during inspiration [19]. This indicated that afferent reactions from skeletal muscle can inhibit cardiac parasympathetic tone. Mechanically sensitive group III skeletal muscle afferents are known to be robustly activated at the onset of electrically evoked muscle contraction [48]. Therefore, the rapidity of the cardiac autonomic responses described above suggests a predominant role for these afferents. Thus, we supposed that synchronization between the motor and the expiration phases could enhance autonomic response to physical exercise, particularly through the inhibition of parasympathetic control by type III afferents. On the contrary, Matsumoto et al. (2011) observed a suppression of sympathetic activity and an activation of parasympathetic control during incremental ergometric exercise in healthy volunteers when the expiration phase was extended and maintained at 4 s during the effort [49]. Our work is in line with this latest study showing an interaction between movement and breathing, particularly expiration and the potential implication of autonomic reflexes in cardiovascular diseases [50].

For all these reasons, we speculate that engaging in rhythmic physical exercise, synchronizing the passive phase with expiration and by promoting a slow-paced breathing, may help limit arrhythmias in at-risk populations. Further studies are still needed to confirm this effect in clinical populations.

### Limitations and perspectives

The main limitation of this work is related to our small sample size: a larger sample size would have enhanced the ability to generalize the findings. Moreover, this approach should be specifically studied in patients with chronic cardiovascular diseases, as it holds the greatest clinical relevance for them. Also, we observed no muscle activation via EMG under passive movement conditions such as in previous studies [51], [52]. However, during passive movement without motor command activation, we cannot definitively rule out spinal activation. Indeed, muscle stretch reflexes or joint position-sensitive reflexes may trigger subtle activations [53]. Moreover, we observed that the amplitude of movement appears significantly different depending on the expiration timing (1.5°), but this is not the case for neuromuscular activation (EMG). The greater parasympathetic modulation when expiration is synchronized on return cannot therefore be attributed to a lower central activation. Nevertheless, these hypotheses would have a negligible effect on our cardiac autonomic responses unless they interact with the cardiac autonomic responses we have highlighted as in previous reports [54]. Finally, In our study, as many others investigating slow-paced breathing, we did not monitor end-tidal carbon dioxide, despite its fundamental role in autonomic regulation associated with respiratory control [5]. Although Uryga et al. (2025) recently showed that slow-paced breathing at 6 cycles per minute has no effect [55], and that an isolated passive or active movement induces the same level of end-tidal carbon dioxide, as demonstrated by Salinet et al. (2013), we cannot exclude the possibility that modulations of end-tidal carbon dioxide may vary under our experimental conditions and account for the observed changes [56]. These changes could therefore represent one of the mechanisms involved in the responses we observed, but further studies will be needed to determine this by monitoring end-tidal carbon dioxide.

## Conclusion

Central motor control plays an important and primary role in autonomic cardiac modulations and parasympathetic withdrawal in a simple, isolated joint movement. Slow-paced breathing favors parasympathetic control compared to pace- or spontaneous breathings but was disturbed by voluntary active movements. Finally, expiration during the return of dynamic exercise may preserve parasympathetic regulation. Further studies are needed to better understand these mechanisms and to study these responses in patients who suffer from chronic diseases.

## Ethical approval

All procedures performed in studies involving human participants were in accordance with ethical standards of the institutional research committee.

## CRediT authorship contribution statement

**Florian Chouchou:** Writing – original draft, Validation, Supervision, Software, Formal analysis, Conceptualization. **Chantal Verkindt:** Writing – review & editing, Supervision, Project administration, Conceptualization. **Laurent Reale:** Writing – review & editing, Software, Data curation. **Antoine Mariani:** Writing – review & editing, Software, Methodology, Formal analysis. **Pauline Doussineau:** Writing – original draft, Methodology, Formal analysis, Data curation.

## Informed consent

Informed consent was obtained from all individual participants included in the study.

## Declaration of Competing Interest

The authors declare the following financial interests/personal relationships which may be considered as potential competing interests: DOUSSINEAU reports financial support was provided by Région La Réunion. If there are other authors, they declare that they have no known competing financial interests or personal relationships that could have appeared to influence the work reported in this paper.

## Data Availability

The datasets used or analyzed during the current study are available from the corresponding author on reasonable request.
